# Feedback of patient survey on medication improves the management of polypharmacy: a pilot trial

**DOI:** 10.1186/s12875-021-01396-x

**Published:** 2021-02-22

**Authors:** Yuta Hirose, Kiyoshi Shikino, Yoshiyuki Ohira, Sumihide Matsuoka, Chihiro Mikami, Hayami Tsuchiya, Daiki Yokokawa, Akiko Ikegami, Tomoko Tsukamoto, Kazutaka Noda, Takanori Uehara, Masatomi Ikusaka

**Affiliations:** 1 Department of General Medicine, Chiba University Hospital, 1-8-1, Inohana, Chuo-ku, Chiba-city, Chiba pref Japan; 2 Department of General Medicine, International University of Health and Welfare, Narita, Japan; 3 Minamihama Clinic, Funabashi, Japan

**Keywords:** Feedback, Polypharmacy, Questionnaire, Health professions

## Abstract

**Background:**

Patient awareness surveys on polypharmacy have been reported previously, but no previous study has examined the effects of sending feedback to health professionals on reducing medication use. Our study aimed to conduct a patient survey to examine factors contributing to polypharmacy, feedback the results to health professionals, and analyze the resulting changes in the number of polypharmacy patients and prescribed medications.

**Methods:**

After conducting a questionnaire survey of patients in Study 1, we provided its results to the healthcare professionals, and then surveyed the number of polypharmacy patients and oral medications using a before-after comparative study design in Study 2. In Study 1, we examined polypharmacy and its contributing factors by performing logistic regression analysis. In Study 2, we performed a t-test and a chi-square test.

**Results:**

In the questionnaire survey, significant differences were found in the following 3 items: age (odds ratio (OR) = 3.14; 95% confidence interval (CI) = 2.01–4.91), number of medical institutions (OR = 2.34; 95%CI = 1.50–3.64), and patients’ difficulty with asking their doctors to deprescribe their medications (OR = 2.21; 95%CI = 1.25–3.90). After the feedback, the number of polypharmacy patients decreased from 175 to 159 individuals and the mean number of prescribed medications per patient decreased from 8.2 to 7.7 (*p* < 0.001, respectively).

**Conclusions:**

Providing feedback to health professionals on polypharmacy survey results may lead to a decrease in the number of polypharmacy patients. Factors contributing to polypharmacy included age (75 years or older), the number of medical institutions (2 or more institutions), and patients’ difficulty with asking their physicians to deprescribe their medications. Feedback to health professionals reduced the percentage of polypharmacy patients and the number of prescribed medications.

**Trial registration:**

UMIN. Registered 21 June 2020 - Retrospectively registered, https://www.umin.ac.jp/ctr/index-j.htm

**Supplementary Information:**

The online version contains supplementary material available at 10.1186/s12875-021-01396-x.

## Background

Polypharmacy is a situation in which a patient takes multiple oral medications. It is known to increase the risk of adverse drug events and the hospitalization rate and cause a decline in adherence to treatment as well as a deterioration in quality of life (QOL) [1–3]. In addition, calculations have shown that in Japan, pharmaceuticals worth a total of 50 billion Japanese yen a year are destroyed and discarded without being used because of polypharmacy [4], which has contributed to increased medical costs. Thus, addressing polypharmacy is an urgent issue.

The reduction of medication use in polypharmacy patients is known to improve the quality of medical care [5]. Standard criteria currently used for the detection of inappropriate prescriptions among older adults include the Beers Criteria [6] in the United States and the STOPP/START Criteria [7] in Europe. The guideline for safe drug therapy for older adults [8], which uses a modified version of the STOPP criteria, has developed into a standard, reflecting current drug treatment in Japan. Measures against polypharmacy have been taken, for example, use of the medication booklet to identify prescription drugs, assess adherence, and recommend non-pharmacological treatments.

Patient awareness surveys on polypharmacy have been reported previously [9, 10], but no previous study has examined the effects of sending feedback to health professionals on reducing medication use. Our study aimed to conduct a patient awareness survey to examine the factors contributing to polypharmacy (Study 1) and elucidate changes in the number of polypharmacy patients and in the number of prescribed medications that result from sharing survey results as feedback to health professionals (Study 2).

## Methods

### Study 1

#### Participants and setting

The study was conducted on patients aged 20 years or older who consulted Minamihama clinic, general outpatient department in July 2016 for regular drug prescriptions. Minamihama clinic is in a city that is approximately 20 min from Tokyo, with a population of roughly 600,000. The clinic is run by five doctors and provides primary care services, including outpatient consultations, dialysis, and home visits.

#### Procedure

In an awareness survey on polypharmacy, the participants answered a self-administered questionnaire during their regular visits at the clinic. The questionnaire was given to all eligible patients in the study period. Eligible patients received a questionnaire from the medical office at the reception desk and completed it while waiting. After filling it out, they submitted it to the collection box. Questionnaires were given to consecutive patients.

The exclusion criteria were: patients who were under 20 years of age, institutionalized, or receiving care via specialized outpatient consultations, dialysis, or in-home visits. In addition, we considered questionnaires incomplete and excluded them if respondents had failed to answer one or more of their items.

#### Questionnaire

There were 7 questionnaire items (supplementary 1). The questions asked: the patient’s age, gender, number of medical institutions regularly consulted, whether the patient felt a need for prescription drugs, whether they understood the reason for the prescriptions, whether deprescribing medications made them anxious, and whether the patient had difficulty with asking their physicians to deprescribe their medications. All questions, except those about age, gender, and the number of medical institutions regularly consulted, had two answer options, “yes” or “no.” If patients answered yes to whether they felt a need for prescription drugs, it was interpreted that all drugs are necessary, and if patients answered yes to whether they understood the reason for the prescriptions, it was interpreted that patients understood the reason for taking all drugs. We determined the survey items based on focus group discussions and the previously validated Patients’ Attitudes Towards Deprescribing (PATD) questionnaire, which gauges how patients feel about their prescription drugs [3] (Y.H, K. S, Y. O, S. M, M. C, T.H). The questionnaire used in our study was developed for this study.

#### Analytic methods

We followed an observational study design, and we conducted it in accordance with the Strengthening the Reporting of Observational Studies in Epidemiology (STROBE) guidelines. In Japan, taking 6 or more oral medications has been reported to increase the frequency of adverse drug events [11]. In our study, polypharmacy was defined as taking 6 or more oral medications. Therefore, patients taking 6 or more oral medications were assigned to a polypharmacy group, and those taking 5 or less oral medications were assigned to a non-polypharmacy group. The number of oral medications taken by each patient was confirmed by a researcher (YH) who used electronic health records. We performed univariate and binomial logistic regression analyses, using a t-test and a chi-square test, to examine factors contributing to polypharmacy. The statistical power was set to 80%, the level of significance was set to 0.05, and a sample size of 134 people was necessary to demonstrate a significant difference.

All analyses were performed using the SPSS Statistics for Windows 26.0 software package (IBM, Armonk, USA).

### Study 2

#### Participants and setting

The study was conducted among patients aged 20 years or older who regularly visited the Minamihama clinic during 1 month prior to and 1 month after we sent feedback to health professionals in August 2016. There were no staff changes during the above-mentioned periods.

#### Intervention

We provided feedback to all 12 healthcare professionals (5 doctors, 4 nurses, 2 pharmacists, and 1 social worker) working at the clinic. For 15 min, we disclosed the results of the questionnaire survey from Study 1. Feedback was given face-to-face as a group.

Immediately after providing the results, we presented the 12 health professionals with 3 questions. We asked them: were the survey results useful for understanding the current state of polypharmacy patients, would there be changes in the medical care they provided now that they knew the survey results; and were the survey results unexpected (Table 1). For those who responded “Yes” to the question, “Were the survey results unexpected?”, the health professionals’ answer “more than expected,” “as expected,” or “less than expected.” as the next item. Items were the results obtained from Study1 regarding percentage of polypharmacy, and the questionnaire items (“Do you feel that prescriptions are necessary?”, “Do you understand the reason for prescription?”, “Do you have anxiety about reducing medicine?”, and “Do you feel difficulty talking about reducing medicine?”) (Table 1).

**Table 1:** Interview with survey results feedback

**1 Are the survey results useful for understanding the current state of polypharmacy patients?**	
**2 Will the medical care you provide change now that you know the survey results?**	
**3 Were the survey results unexpected?**	
**(1) Percentage of polypharmacy patients**	
**(2) Felt prescriptions were necessary**	
**(3) Understanding the reason for the prescription**	
**(4) Anxiety about reducing medicine**	
**(5) Difficulty talking about reducing medicine**	

Question 1, 2, 3:Answer Yes or No.

Question (1), (2), (3), (4), (5): Answer “more than expected,” “as expected,” or “less than expected”

#### Outcome and measures

As primary outcomes, the number of polypharmacy patients and the number of prescribed medications were measured in adult patients who visited Minamihama clinic regularly during a one-month period before and after we gave feedback (April and October 2016). To determine the number of prescribed medications, medical receipt data was surveyed, and participants taking 6 or more oral medications were considered polypharmacy patients.

#### Analytic methods

We followed a before-after comparative study design. We surveyed the number of polypharmacy patients and the number of oral medications before and after feedback, and we performed analyses using a t-test and a chi-square test. The statistical power was set to 80%, the level of significance was set to 0.05, and a sample size of 113 individuals was necessary in each measurement in order to demonstrate significant differences.

All analyses were performed using the SPSS Statistics for Windows 26.0 software package (IBM, Armonk, USA). With the α error estimated at 0.05 and β error at 0.2 (with the power of detection at 0.8), the minimum sample size necessary to compare the difference between the pre-intervention and post-intervention group data was 150 participants for this study.

## Results

### Study 1:a questionnaire survey of patients

**Fig. 1:**
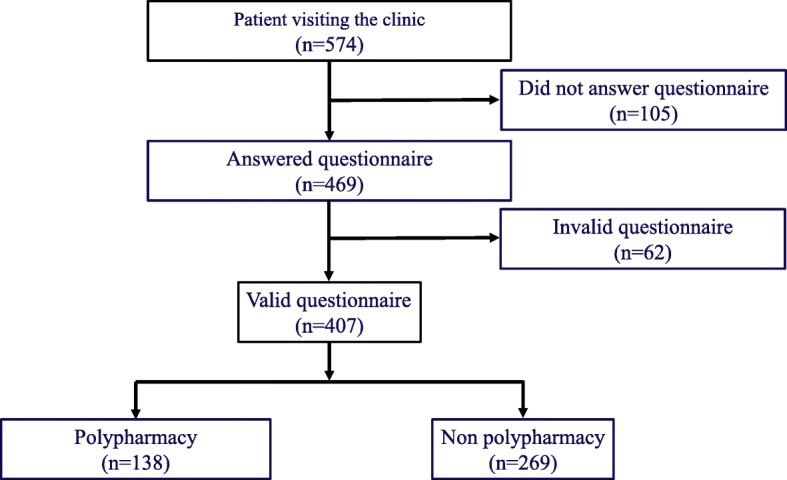
Outline of Study 1. The number of patients who had medical care consultations was 574, the number of survey respondents was 469 (81.7%), among them 407 individuals (70.9%) provided valid responses. We compared the polypharmacy group, which was composed of 138 participants (33.9%), with the non-polypharmacy group, which was composed of 269 participants

The number of patients who had medical care consultations was 574, the number of survey respondents was 469 (81.7%), among them 407 individuals (70.9%) provided valid responses. We compared the polypharmacy group, which was composed of 138 participants (33.9%), with the non-polypharmacy group, which was composed of 269 participants (66.1%; Fig. 1). For age and the number of medical institutions visited on a regular basis for medical care, the cutoff values were set based on the receiver operating characteristic (ROC) curve; the cutoff value for age was 75 years (area under the curve (AUC) = 0.690; 95% confidence interval (CI) = 0.64–0.74, *p* < 0.001), and the cutoff value for the number of medical institutions visited on a regular basis was 2 (AUC = 0.640; 95% CI = 0.58–0.70, *p* < 0.001). Univariate analysis showed that the percentages of the following items were significantly higher in the polypharmacy group than in the non-polypharmacy group: age 75 years or older, 2 or more medical institutions visited on a regular basis, and “patients’ difficulty with telling physicians about their wish to reduce their medication use” (Table 2). In the multivariate analysis using a binomial logistic regression analysis, according to the best-subset selection procedure, the following 7 items were entered: age, gender, number of medical institutions visited on a regular basis, the questions “Do you feel that the prescribed medications were necessary,” “Do you understand the reasons why the medications were prescribed to you,” “Would you be worried if your medications were deprescribed,” and “Is it difficult to ask your doctor to deprescribe your medications.” The following 3 items were extracted as factors contributing to polypharmacy: age (75 years or older; odds ratio (OR) = 3.14; 95% CI = 2.01–4.91), number of medical institutions visited on a regular basis (2 or more institutions; OR = 2.34; 95%CI = 1.50–3.64) and difficulty with asking their doctors to deprescribe their medications (OR = 2.21; 95%CI = 1.25–3.90; Table 3).

**Table 2:** Factors Influencing Polypharmacy (Simple Correlations)

	Polypharmacy group (***n*** = 138)	Non polypharmacy group (***n*** = 269)	***p***-value
**Age ≧ 75, n (%)**	**76 (55.1)**	**72 (26.8)**	**< 0.001**
**Male, n (%)**	**70 (50.7)**	**150 (55.8)**	**0.334**
**Number of family doctors ≧ 2, n (%)**	**81 (58.7)**	**92 (34.2)**	**< 0.001**
**Feeling prescriptions were necessary, n (%)**	**111 (80.5)**	**195 (72.1)**	**0.079**
**Understanding reason for prescriptions, n (%)**	**125 (90.6)**	**252 (93.7)**	**0.257**
**Anxiety about reducing medicine, n (%)**	**66 (47.8)**	**165 (39.0)**	**0.089**
**Difficulty talking about reducing medicine, n (%)**	**34 (24.6)**	**33 (12.3)**	**0.001**

Univariate analyses showed significant differences between the polypharmacy group and the non-polypharmacy group in terms of the following 3 items: age, medical institutions visited for medical care, and the difficulty that patients had with asking their doctors to deprescribe their medications

**Table 3:** Factors Influencing Polypharmacy (Multiple Logistic Regression Analysis)

Items	OR (95%CI)	***p***-value
**Age (75 or over)**	**3.14 (2.01–4.91)**	**< 0.001**
**Number of family doctors (2 or more)**	**2.34 (1.50–3.64)**	**< 0.001**
**Difficulty talking about reducing medicine**	**2.21 (1.25–3.90)**	**0.006**

In a multivariate analysis using binomial logistic regression analysis, significant differences were found in the following 3 items: age, medical institutions visited for medical care, and the difficulty that patients had with asking their doctors to deprescribe their medications

*OR* Odds ratio, *95% CI* 95% confidence interval

### Study 2: feedback of survey results and interview to health professionals and before-after scrutiny of medical receipt data for polypharmacy

In total, 814 patients visited the clinic before and 791 after we gave our feedback to the health professionals. The patients who visited the clinic both before and after our feedback accounted for 592 individuals. Among them, patients who fell under the category of polypharmacy before feedback accounted for 175 individuals (29.8%). Those who did so after feedback accounted for 159 individuals (26.8%). Therefore, the proportion had decreased significantly after feedback (*p* < 0.001; Table 4). In addition, the number of prescribed medications per patient was 8.2 before the intervention, and it significantly decreased to 7.7 after the intervention (*p* < 0.001; Table 4).

**Table 4:** Outcomes: The number of oral medications and the number of polypharmacy patients

	Pre-intervention	Post-intervention	***p*** value
**Oral medication, number (SD)**	**8.2 (2.2)**	**7. 7 (2.2)**	**< 0.001**
**Polypharmacy patients, number**	**175**	**159**	**< 0.001**

Among the 592 patients who visited the clinic both before and after intervention, comparison between data from before and after the intervention showed that the number of polypharmacy patients and the number of prescribed medications decreased significantly after the intervention

From the interview results with the 12 health professionals (5 doctors, 4 nurses, 2 pharmacists, and 1 social worker), all respondents answered “Yes” (12 answered “Yes,” and no one answered “No”) to the following questions: “Are the survey results useful for understanding the current state of polypharmacy patients,” “Will the medical care you provide change now that you know the survey results,” and “Were the survey results unexpected” (Table 5). Regarding the percentage of polypharmacy and the question of whether the patients understood the reasons why the medications were prescribed, the majority of the health professionals answered that the findings were more than they had expected (58.3 and 91.6%, respectively). As for the items “Do you feel that the prescribed medications were necessary,” “Would you be worried if those medications were deprescribed,” and “Is it difficult to ask your doctor to deprescribe your medications,” the majority answered that the findings were as expected (50.0, 58.3, and 58.3%, respectively; Table 5).

**Table 5:** Results: Interview following survey results feedback

	Yes, n (%)	No, n (%)	
**1 Are the survey results useful for understanding the current state of polypharmacy patients?**	12 (100)	0 (0)	
**2 Will the medical care you provide change now that you know the survey results?**	12 (100)	0 (0)	
**3 Were the survey results unexpected?**	12 (100)	0 (0)	
	More than expected, n (%)	As expected, n (%)	Less than expected, n (%)
**(1) Percentage of polypharmacy patients**	7 (58.3)	4 (33.3)	1 (8.4)
**(2) Felt prescriptions were necessary**	4 (33.3)	6 (50.0)	2 (16.7)
**(3) Anxiety about reducing medicine**	3 (25.0)	7 (58.3)	2 (16.7)
**(4) Understanding the reason for the prescription**	11 (91.6)	1 (8.4)	0 (0)
**(5) Difficulty talking about reducing medicine**	1 (8.4)	7 (58.3)	4 (33.3)

Results of the interviews with the 12 health professionals. Participants who answered Yes to the question “Were the survey results unexpected” were instructed to respond to questions (1) to (5) by choosing either “more than expected,” “as expected,” or “less than expected”

## Discussion

Study 1 showed that the following three items were contributing factors to polypharmacy: age (75 years or older), the number of medical institutions visited on a regular basis for medical care (2 different locations or more), and patients’ difficulty with asking their doctors to deprescribe their medications. The finding that age is a risk factor for polypharmacy was consistent with previous reports [12]. In Japan, the number of patients with multimorbidities have increased as population aging has advanced [13]. Reports from previous studies conducted in the United States and Europe have shown that the percentage of polypharmacy is high in older adults because of coexisting chronic diseases [14–17]. Previous reports have shown that the risk of polypharmacy is higher when the number of prescribing physicians is large [18]. In Japan, the health insurance system has enabled free access to medical institutions [19]. As a result, most patients visit multiple medical institutions [20] and receive prescriptions from several physicians. There is a lack of cooperation between medical institutions, which are unaware of each other’s prescriptions. This situation promotes “prescription cascades” in which similar drugs are prescribed more than once [21], and the number of prescribed medications increases. There have been no previous reports on the difficulty that patients have with asking their doctors to deprescribe their medications; it is a newly identified risk factor for polypharmacy. Asking to deprescribe medications can impact the relationship between the patient and the physician in some cases [22], which may have been the reason why it presented a challenge for some respondents. Health professionals need to actively confirm polypharmacy patients’ intentions. They should ask them whether they wish their physician to deprescribe their medications. Thereafter, health professionals need to work on deprescribing the medications that the patients wish to reduce.

Study 2 showed that when feedback on the risk factors for polypharmacy was sent to health professionals, the proportion accounting for the polypharmacy group decreased, and the number of prescribed medications declined. Previous reports have shown that the promotion of changes in consciousness (aimed at improving the quality of medical care) through sending feedback to health professionals has led to an improvement in the quality of medical care [23]. In the questionnaire survey results on the feedback submitted to healthcare professionals in our study, all respondents answered that a survey of the current situation regarding polypharmacy was useful, and answered that after hearing the results of the survey, they would implement changes in the medical care they provide. In addition, all respondents answered that the results of the questionnaire survey conducted in Study 1 were unexpected. Many respondents answered the percentage of polypharmacy patients was higher than expected, suggesting a change in awareness of polypharmacy before and after the feedback. Health professionals make behavioral changes in medical care (e.g., considering risk of drug-induced harm in determining the required intensity of deprescribing intervention, assessing whether the drug is necessary based on risks and benefits, and deprescribing less useful drugs) [24]. This may lead to a decrease in the number of polypharmacy patients. Nearly all respondents answered that more patients understood the reasons for prescribing than expected. In Study 1, more than 90% of patients answered that they understood the reasons for prescribing, which could be interpreted as health professionals continued to prescribe to patients while thinking they did not understand the reasons for prescribing. It is also possible that patients had an incorrect self-interpretation of the reasons for prescribing. Sharing the reasons for prescribing between health professionals and patients can lead to the discontinued use of less important drugs and improve adherence of important drugs.

Conducting a fact-finding survey of polypharmacy and submitting the results as feedback promotes changes in consciousness and behavior among health professionals [23] and may reduce the percentage of polypharmacy. Martin et al. [25] explained that following a method consisting of submitting a written opinion (from a pharmacist to a physician) regarding oral medications and giving patients pamphlets on polypharmacy, the number-needed-to-treat (NNT) was 3.22 for reducing medication by one drug. In our study, the NNT was 71. Therefore, the intervention conducted in our study was not as efficient as those in previous studies. However, the method we undertook is inexpensive, can be performed in any type of medical institution, and based on written opinions, is easier to do than the interventions mentioned in previous studies [25]. Thus, our method can be expected to yield beneficial effects when carried out consistently in routine medical care.

Instead of relying on physicians alone, intervention through multi-sectoral collaboration involving nurses, pharmacists, and social workers is important to eliminating polypharmacy [26]. Physicians can assess the prescriptions; nurses can ask patients about their oral medications and submit reports to the treating physicians; pharmacists can intervene by answering questions (regarding drug prescriptions), and social workers can monitor the condition of polypharmacy patients receiving nursing care and submit reports to the treating physicians.

Our study has several limitations. The study was conducted in a single clinic, and it remains unverified that the data can be used in other facilities with a different medical care setting. To check its validity, an additional study will need to be conducted at multiple facilities. The second limitation of our research is that it was designed as a pre- and post-intervention study. The change in the percentage of polypharmacy may have been due to confounding factors beyond our provision of feedback on the questionnaire survey results to the healthcare professionals. The third limitation is the questionnaire used here itself has not been validated. The fourth limitation is that there was no way to confirm long-term behavior modification among the health professionals regarding polypharmacy. A follow-up study needs to be conducted to determine the period within which the residual effects of a single intervention can be expected and whether repeating the intervention could serve as a “booster” for decreasing polypharmacy.

## Conclusion

Factors contributing to polypharmacy included age (57 years or older), the number of medical institutions visited on a regular basis (2 or more institutions), and patients’ difficulty with asking their doctors to deprescribe their medications, which, importantly, was previously unreported. Moreover, sending health professionals feedback on the factors contributing to polypharmacy led to changes in awareness among health professionals. It may also lead to a decrease in the percentage of polypharmacy patients and in the number of prescribed medications.

## Supplementary Information

**Additional file 1.** Prescription Drug Questionnaire.

## Data Availability

Not applicable.

## Abbreviations

AUC: Area under the curve
NNT: Number-needed-to-treat
PATD: Patients’ Attitudes Towards Deprescribing
QOL: Quality of life
ROC: Receiver operating characteristic
STROBE: Strengthening the Reporting of Observational Studies in Epidemiology
