# Supplementary material for: Feedback of patient survey on medication improves the management of polypharmacy: a pilot trial
Source: BMC Fam Pract. 2021 Feb 22;22:42. doi: 10.1186/s12875-021-01396-x (PMC7901107; doi:10.1186/s12875-021-01396-x)
Supplement: Supplementary file 1 — Additional file 1. Prescription Drug Questionnaire. [file 12875_2021_1396_MOESM1_ESM.docx]

Prescription Drug Questionnaire

We are conducting a survey regarding regular prescriptions.

We would be very grateful if you could fill out this questionnaire.

Age：　　 year old

Gender：Male・Female

Number of family doctors：

Number of oral medicine：

Please answer the following questions with "yes" or "no".

1. Do you feel that prescriptions are necessary? Yes・No
2. Do you understand the reason for prescription? Yes・No
3. Do you have anxiety about reducing medicine? Yes・No
4. Do you feel Difficulty talking about reducing medicine? Yes・No
